# Fermi surface topology in a metallic phase of VO_2_ thin films grown on TiO_2_(001) substrates

**DOI:** 10.1038/s41598-018-36281-8

**Published:** 2018-12-17

**Authors:** Yuji Muraoka, Hiroki Nagao, Yuichiro Yao, Takanori Wakita, Kensei Terashima, Takayoshi Yokoya, Hiroshi Kumigashira, Masaharu Oshima

**Affiliations:** 1 Research Institute for Interdisciplinary Science, Okayama University, 3-1-1 Tsushima-naka, Tsushima, Kita-ku, Okayama, 700-8530 Japan; 2 Graduate School of Natural Science and Technology, Okayama University, 3-1-1 Tsushima-naka, Tsushima, Kita-ku, Okayama, 700-8530 Japan; 3 High Energy Accelerator Research Organization (KEK), Photon Factory, 1-1 Oho, Tsukuba, Ibaraki, 305-0801 Japan; 4 The Institute for Solid State Physics, The University of Tokyo, 5-1-5 Kashiwanoha, Kashiwa, Chiba, 277-8581 Japan; 5 Present Address: Institute of Multidisciplinary Research for Advanced Materials, Tohoku University, 2-1-1 Katahira, Aoba-ku, Sendai, 980-8577 Japan

## Abstract

Since the first observation of the metal-to-insulator transition (MIT), VO_2_ has attracted substantial attention in terms of whether this transition is impelled by electron–phonon interaction (Peierls transition) or electron–electron interaction. Regarding Peierls transition, it has been theoretically predicted that the Fermi surface (FS) cross-section exhibits certain nesting features for a metallic phase of VO_2_. Various experimental studies related to the nesting feature have been reported. Nevertheless, there is no experimental result on FS topology. In this work, we determine the FS topology of the metallic phase of VO_2_ through studies of VO_2_ epitaxial thin films on TiO_2_(001) substrates, using synchrotron radiation angle-resolved photoemission spectroscopy (ARPES). Three electron pockets around Γ are observed in band structures along the Γ–*X* direction. These three bands form electron surfaces around Γ in the Γ*XRZ* plane. Furthermore, the lowest energy band FS exhibits the nesting feature corresponding to a nesting vector $\overrightarrow{q}$ = Γ*R*, as predicted by the calculation. Our results strongly indicate the formation of the charge-density wave with $\overrightarrow{q}$ = Γ*R* and thus, the importance of Peierls transition for the mechanism of the MIT in VO_2_.

## Introduction

VO_2_ has an electron configuration of 3*d*^1^, and shows a metal–to-insulator transition (MIT) at *T*_MI_ = 341 K^1^. At the *T*_MI_, a structural change occurs from a rutile-type tetragonal structure in the high temperature metallic phase to a monoclinic structure in the low-temperature insulator phase. In the monoclinic structure, V atoms form zig-zag type pairs along the *c* axis. A model of electronic structure was proposed by Goodenough^2^. He started from describing the splitting of the *d*_||_ band and the π* bands in the *t*_2*g*_ states. In the metallic phase the *d*_||_ and π* bands overlap. In the monoclinic phase, the π* band is lifted above the Fermi level (*E*_F_) due to a tilting of the V–V pairs, and the *d*_||_ band splits into a filled bonding band and an empty anti-bonding band caused by the V–V pairing, resulting in opening up an energy gap. The proposed electronic structure was verified by optical^3^ and X-ray photoelectron^4^ measurements. The origin of the *d*_||_ band splitting in the monoclinic insulator phase is to be determined.

There is extensive debate regarding whether the origin of the *d*_||_ band splitting is an electron–phonon interaction (Peierls transition)^5–8^, an electron–electron interaction (Mott transition)^9–13^, or a combination of the two^14–16^. With regard to Peierls transition, Gupta *et al*.^17^ performed a first-principle band calculation using the linear-combination-of-atomic-orbitals (LCAO) method and demonstrated that for the metallic phase of VO_2_, the lowest *d*_||_ band FS exhibits certain nesting features with a nesting vector $\overrightarrow{q}$ = 2$\overrightarrow{k}$_F_ = Γ*R*. Their work indicates that the charge-density waves (CDWs) are significant for the origin of the transition. This prediction was supported by studies of X-ray diffuse scattering^18^. Apart from X-ray studies, various experimental and theoretical studies support the importance of the electron–phonon interaction^19,20^. Notwithstanding these intensive works, the likelihood of Peierls transition underlying the MIT is still under debate. This is because there is no experimental result of FS topology of VO_2_. The deficiency of experimental data renders it difficult to verify whether Peierls transition underlies the MIT.

ARPES is a highly effective method for determining the energy band structures and FS topology in crystalline solids. It has been encouraged to perform the ARPES studies of VO_2_ single crystal. However, till the present, few ARPES measurements of VO_2_ single crystal have been conducted. This is mainly because it is difficult to obtain a chemically stable cleavage plane in the VO_2_ single crystal with the three-dimensional crystal structure. Recently, the usefulness of the epitaxial thin films of VO_2_ for PES measurements has been reported. The VO_2_ thin films grown epitaxially on TiO_2_ (001) substrates were prepared using a pulsed laser deposition (PLD) technique^21^. The MIT temperature of the films was decreased to 300 K. The decrease in the transition temperature is understood as a result of an in-plane tensile strain effect. Angle-integrated PES measurements of the VO_2_ thin films were performed using the Mg *Kα* line and He I resonance line^22^. The results of PES measurements were reproduced and reliable PES spectra of the VO_2_ thin films were obtained. The temperature dependence of the PES spectra for the films was presented. A synchrotron radiation PES study^23,24^ and synchrotron radiation ARPES measurements of VO_2_ epitaxial thin films on TiO_2_(001) were also performed^25^. In the latter, by changing the incident photon energy, a distinct band dispersion of O 2*p* and V3*d* bands along the Γ–*Z* direction was observed for the metallic phase of the VO_2_ thin films. These works motivate us to perform a synchrotron radiation ARPES study of VO_2_ epitaxial thin films on TiO_2_(001) in order to determine the FS topology of the metallic phase of VO_2_.

In this paper, we report the results of synchrotron radiation ARPES measurements of the metallic phase of VO_2_ epitaxial thin films on TiO_2_(001) substrates. At first, the band structures around Γ in the Γ–*X* direction are determined. Then, the FS topology in the Γ*XRZ* plane is elucidated. Finally, the presence of the nesting vector $\overrightarrow{q}$ = Γ*R* is examined.

## Results and Discussion

### Characterization of VO2 thin films on TiO2(001)

**Figure 1:**
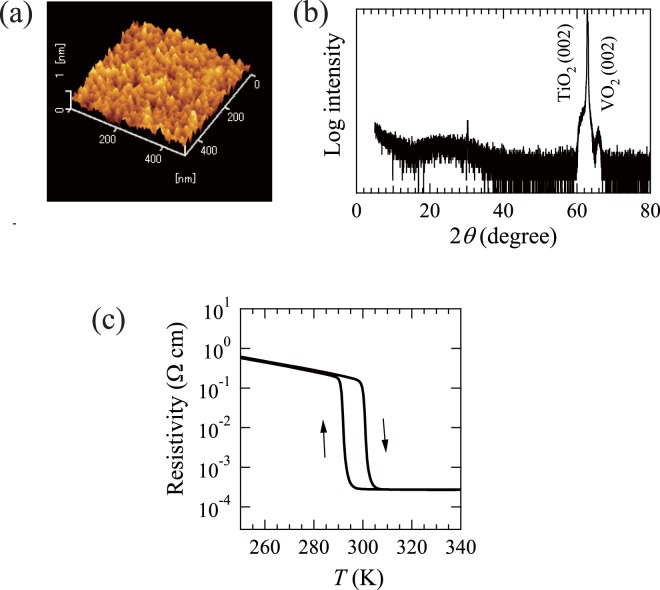
(**a**) AFM image of VO_2_ thin films grown on TiO_2_(001) substrates. The thickness of the VO_2_ thin films was 11 nm. (**b**) XRD pattern of VO_2_ thin films grown on TiO_2_(001) substrates. (**c**) Temperature dependence of resistivity for VO_2_ thin films grown on TiO_2_(001) substrates.

An AFM image of the VO_2_ thin films on TiO_2_(001) is shown in Fig. 1(a). The smooth surface with root mean square of 1.4 Å is observed. An XRD pattern of the prepared films is depicted in Fig. 1(b). The VO_2_(002) peak is observed at approximately 2*θ* = 66°. No other peaks apart from those from VO_2_ and TiO_2_ substrate are detected, indicating the formation of a (001)-oriented single phase for the VO_2_ thin films. The films have the *c*-axis length of 2.844 Å, which is reasonably consistent with that obtained previously^21^. The epitaxy of the films was verified from the X-ray *ϕ* scans of the (202) reflection of the VO_2_ thin films. The films exhibit a four-fold symmetry (four (202) reflection peaks separated by 90°), as is expected in a tetragonal rutile form, indicative of the epitaxial growth of the VO_2_ thin films on the TiO_2_(001) substrates. Fig. 1(c) shows the temperature dependence of the resistivity for the prepared films. The films exhibit distinct MIT with a sharp increase in resistivity by three orders of magnitude at around 300 K. The *T*_MI_ of the films is determined as the midpoint of this increase in the resistivity curve with respect to temperature, and is estimated to be 301 K. The value of *T*_MI_ is lower than the bulk temperature (341 K) owing to an in-plane tensile strain induced by the lattice mismatch between the film and substrate. A steep and large jump in resistivity at the transition indicates the high quality of the films. The results are largely similar to those reported previously^21^, demonstrating the epitaxial growth of the VO_2_ thin films on TiO_2_(001).

### Synchrotron radiation ARPES study of VO2 thin films on TiO2(001)

**Figure 2:**
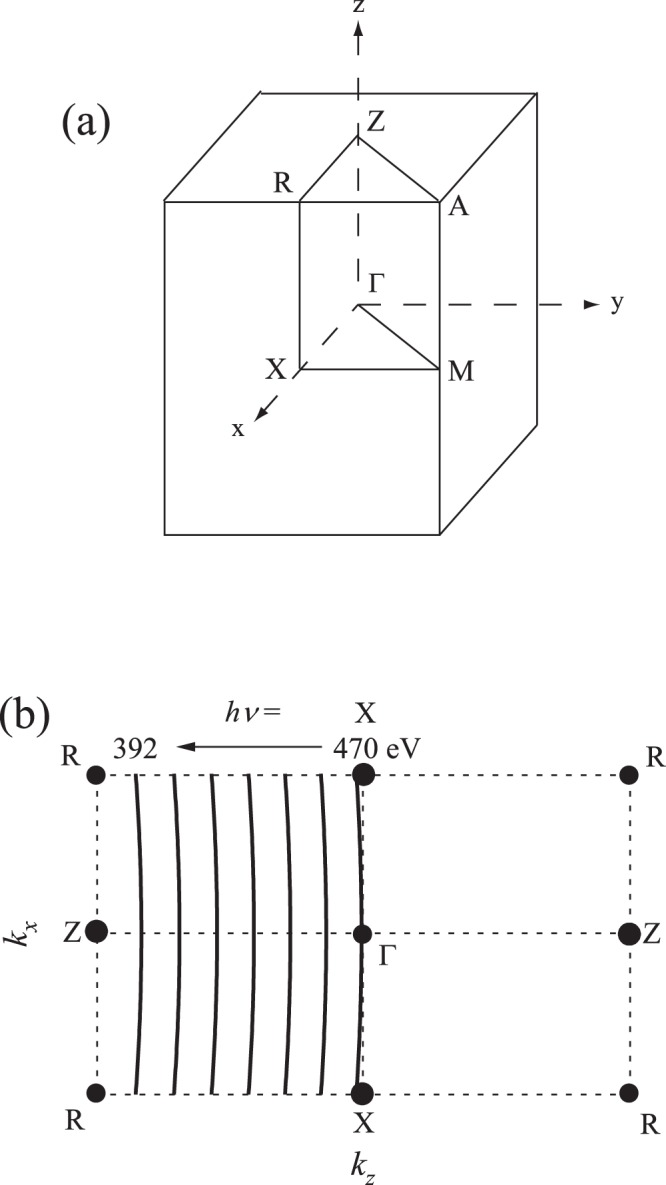
(**a**) Brillouin zone of rutile phase of VO_2_. (**b**) Traces of *k* space in Γ*XRZ* plane for the present ARPES study. Synchrotron radiation ARPES measurements were performed with photon energy values of 470, 457, 444, 431, 418, 405, and 392 eV in the normal emission mode for the metallic phase of the VO_2_ thin films.

Figure 2(a) shows the brillouin zone of the rutile phase of VO_2_. In order to observe the FS in the Γ*XRZ* plane, we performed ARPES measurements by decreasing the incident photon energy from 470 to 392 eV in the normal emission mode for the metallic phase of VO_2_ thin films. The traces in *k* space are shown in Fig. 2(b).

**Figure 3:**
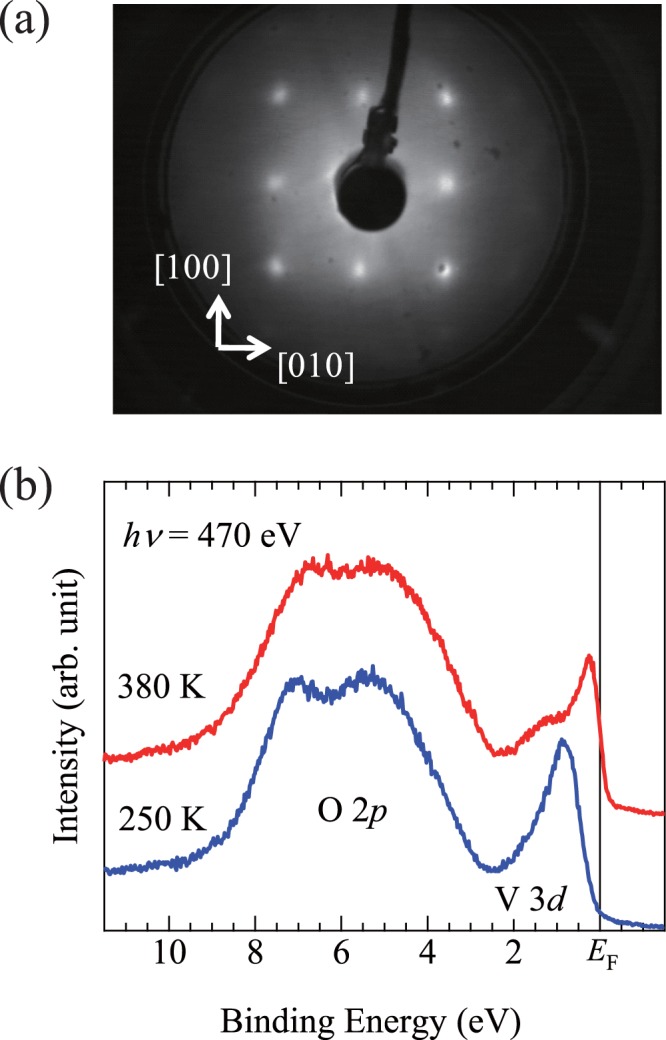
(**a**) LEED pattern of VO_2_(001) surface at incident electron energy of 48 eV. (**b**) Valence-band PES spectra of VO_2_ thin films. The blue line indicates the spectrum of the insulator phase measured at 250 K. The red line indicates the spectrum of the metallic phase measured at 380 K. The spectra were captured at the photon energy of 470 eV.

Prior to ARPES measurements, the films were annealed at 150 °C under a vacuum condition of 5 × 10^−8^ Pa for 10 min to obtain a clean surface. The surface of the VO_2_ thin films was examined by low-energy electron diffraction (LEED). The electron energy was set at 48 eV, where the LEED probing depth is approximately one atomic layer. Figure 3(a) shows the LEED pattern obtained on the VO_2_(001) surface. A clear four-fold symmetry is observed. No additional spot is observed, demonstrating the presence of rutile-type tetragonal surface of the VO_2_(001) films. Figure 3(b) depicts the valence band photoemission spectra of the VO_2_ thin films measured at 250 K for the insulator phase and at 380 K for the metallic phase. A peak located at 0–2 eV is assigned to the V3*d* band and a broad peak located at 3–9 eV is assigned to the O 2*p* band. The shape of the V3*d* band drastically changes through the MIT, whereas the O 2*p* band exhibits no noticeable change. The obtained results are reasonably consistent with those reported previously^4,22–25^. The results of both the LEED image and valence band spectra indicate that the surface of the films is clean enough for obtaining ARPES spectra of VO_2_.

**Figure 4:**
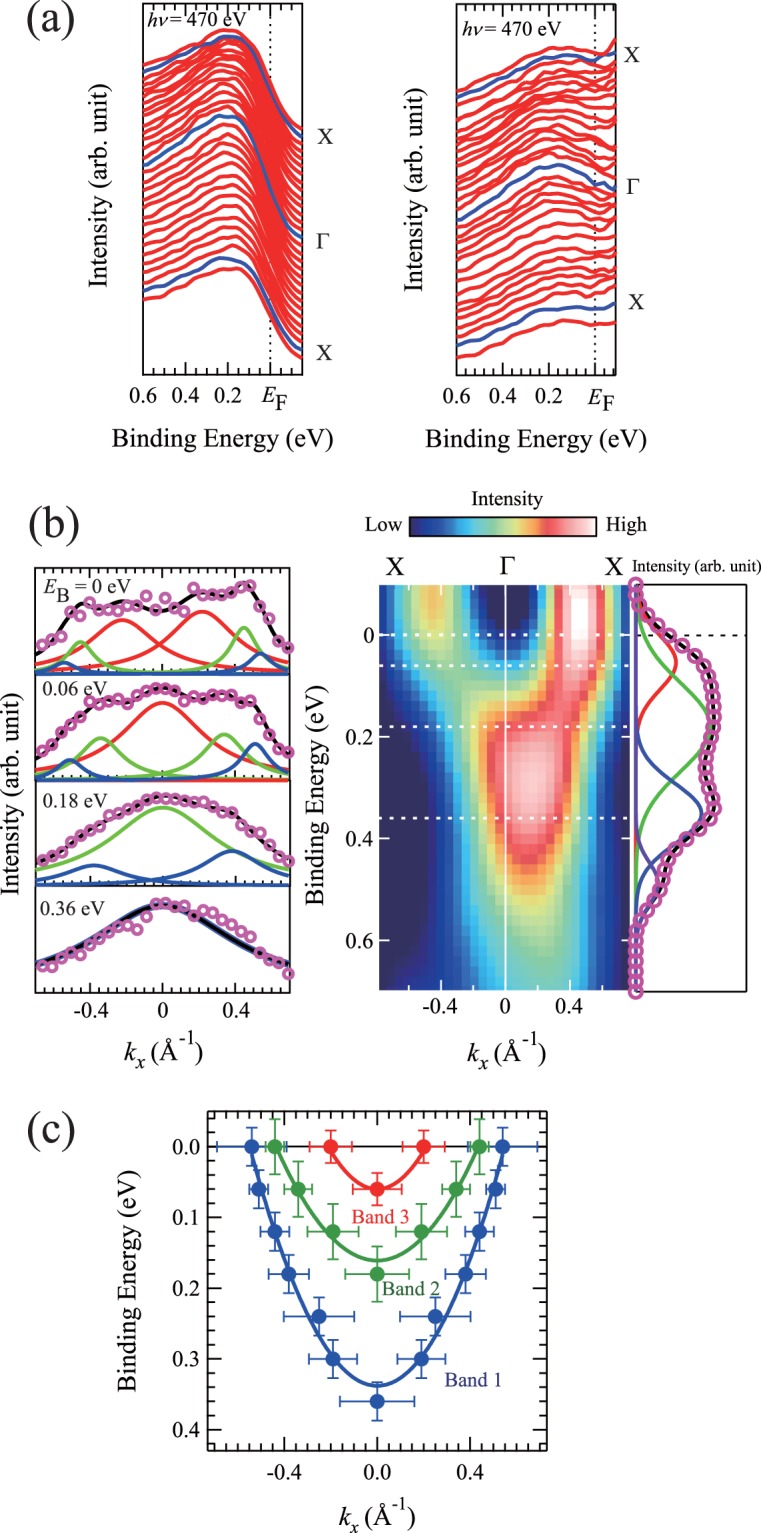
(**a**) (Left) EDCs near *E*_F_ in Γ–*X* direction for VO_2_ thin films grown on TiO_2_(001) substrates. (Right) EDCs divided by the Fermi-Dirac distribution curve convolved by Gaussian up to 3*k*_B_*T*. (**b**) (Middle) Experimental band structure in Γ–*X* direction of VO_2_ thin films grown on TiO_2_(001) substrates. (Left) MDCs at *E*_B_ = 0, 0.06, 0.18, and 0.36 eV of ARPES spectra for VO_2_ thin films on TiO_2_(001) substrates. The results of the curve fittings for the spectrum are also shown. The open circles are experimental data; black lines are the fitting result; and blue, green, and red lines are the components used for the fittings. In the fitting procedure, a Lorentzian function and constant background are used. Each pair of peaks is constrained to have the peak position symmetrical with respect to *k*_x_ = 0 and an equivalent full width at the half maximum. (Right) Second-derivative ARPES spectra with respect to the EDCs at Γ of VO_2_ thin films on TiO_2_(001) substrates. The result of the curve fitting for the spectrum is also shown. In the fitting procedure, a Gaussian function and Shirley background are used. The open circles are experimental data, black line represents the fitting result, and lines colored other than black represent the component used for the fitting. (**c**) Band structures in the Γ–*X* direction for VO_2_ thin films on TiO_2_(001) substrates. The closed circles correspond to the peak position determined by the spectral fitting of the MDCs. The band structures of bands 1, 2, and 3 are represented by the closed blue, green, and red circles, respectively. The solid lines represent the result of the fit to data, assuming parabolic energy dispersions around the Γ point for the three bands. The error bars are defined by the full width at 90% of the maximum of the Lorentzian fits in the horizontal axis and by the full width at 90% of the maximum of the Gaussian fits in the vertical axis. The error bar of *k*_F_ for the band 1 is determined form the peak positions where the fitting results do not reproduce the experimental data well (see Supplementary Fig. S2).

At first, we performed ARPES measurements along the Γ–*X* direction to determine the *d*_||_ bands for the metallic phase of the VO_2_ thin films. This is because the lowest *d*_||_ band provides the nested FS in the band calculation. The photon energy was set to 470 eV to trace around the Γ–*X* high-symmetry line. Figure 4(a) shows the energy distribution curves (EDCs) near the Fermi energy *E*_F_ in the Γ–*X* direction for the metallic phase of the VO_2_ thin films. EDCs divided by the Fermi-Dirac distribution curve convolved by Gaussian are also depicted. Peak structures are observed, attributed to the V3*d* states. The V3*d* bands appear to display energy dispersion. In order to make the peak structure clearer, the Laplacian was determined after being divided by the Fermi function^26^. The result is shown as the intensity plot at the middle panel in Fig. 4(b). It is evident that the V3*d* bands display a distinct concave curve crossing *E*_F_ along the Γ–*X* direction. This indicates the presence of electron pockets around Γ in the Γ–*X* direction.

To obtain further information, we performed the spectral fitting of the momentum distribution curves (MDCs) at different binding energy (*E*_B_) values. The left panel in Fig. 4(b) shows the results of the spectral fitting for the MDCs at *E*_B_ = 0, 0.06, 0.18, and 0.36 eV. At *E*_B_ = 0 eV, two pairs of peaks are not adequate to reproduce the experimental data. Three pairs of peaks are necessary to fit the data correctly. These three pairs of peaks change their positions systematically with varying *E*_B_. At *E*_B_ = 0.06 eV, all the peaks shift toward the center position, and an inner pair of peaks merges. A shoulder structure at 0.5 Å^−1^ is observed. At *E*_B_ = 0.18 eV, the inner peak shifts above *E*_F_, and a middle pair of peaks merges. At *E*_B_ = 0.36 eV, the middle peak disappears, and an outer pair of peaks finally merges. The results indicate the presence of three electron pockets around Γ in the Γ–*X* direction for the V3*d* states. The energy of *E*_B_ = 0.36, 0.18, and 0.06 eV correspond to the bottoms of the outer, middle, and inner electron pockets, respectively. The energy position of the bottoms of the bands is evaluated also by analyzing the ARPES spectrum at Γ. In order to make the peak structure clearer, the second derivative with respect to the EDCs was determined after being divided by the Fermi function, as shown in the right panel of Fig. 4(b). The ARPES intensity curve at Γ can be fitted using four components. Among these, three components exhibit the energy position of *E*_B_ = 0.06, 0.18, and 0.36 eV. The results are reasonably consistent with the result of the MDC analysis, supporting the result indicating the presence of three electron pockets around Γ in the Γ–*X* direction. A remaining component has the energy position of *E*_B_ = 0.49 eV. Considering the results of the MDC analysis, this component does not cut *E*_F_. We speculate that the remaining component originates from the surface one. Hereafter, we focus on these three electron bands and represent the outer electron pocket as band 1, the middle one as band 2, and the inner one as band 3.

In order to observe the band structures along the Γ–*X* direction, the peak positions determined from the spectral fitting of MDCs are plotted as functions of energy and momentum. The results are shown in Fig. 4(c). Three types of electron pockets around Γ are clearly observed. Assuming parabolic energy dispersions around Γ for the three bands, the Fermi momentum (*k*_F_) is determined to be 0.54, 0.44, and 0.21 Å^−1^ for bands 1, 2, and 3, respectively. An occupied bandwidth *W* is estimated to be 0.34, 0.16, and 0.06 eV for bands 1, 2, and 3, respectively. The effective-mass ratio m*/*m*_0_ is determined to be 3.4, 4.5, and 2.8 for bands 1, 2, and 3, respectively. Band 1 is the largest electron pocket and band 3 is the smallest electron pocket among the three. The results are tabulated in Table 1, together with those of the calculation^17^ wherein the Fermi surface nesting feature is predicted.

**Table 1:** Fermi momentum (*k*_F_), occupied bandwidth *W*, and effective-mass ratio m*/*m*_0_ for bands 1, 2, and 3 determined under the assumption of the parabolic energy dispersions around the Γ point for the three bands. The *k*_F_ and *W* of *d*_||_ band estimated from ref.^17^ are also shown for comparison.

	Band	*k*_F_ (Å^−1^)	Occupied bandwidth *W* (eV)	Effective mass ratio m*/*m*_0_
This work	Band 1	0.54	0.34	3.4
	Band 2	0.44	0.16	4.5
	Band 3	0.21	0.06	2.8
LCAO^17^	Outer *d*_||_ band*	0.53	0.45	
	Inner *d*_||_ band*	0.48	0.40	

*The *k*_F_ and *W* of outer and inner *d*_||_ bands are estimated from Figs 3, 8, and 9 in ref.^17^.

According to the calculation, there are two electron bands assigned to *d*_||_ bands in the Γ–*X* direction. Of the two, the lowest *d*_||_ band possesses the nesting feature in the FS topology in the ΓXRZ plane. When comparing the experimental results with the calculation, we can assign band 1 to the lowest *d*_||_ band in the calculation because of the similarity of *k*_F_ and *W*. Band 2 can also be assigned to another *d*_||_ band in theory considering the similarity of *k*_F_, whereas *W* is rather small. However, band 3 is not observed in the calculation. This difference is not severe because more than two electron pockets are likely when the number of *t*_2g_ orbits in V3*d* states is taken into consideration. A small size of electron pockets around Γ is revealed in addition to two *d*_||_ bands in other calculations^20,27^. It is likely that band 3 is attributed to a π* band in V3*d* states because it exhibits a relatively small *W* and m*/*m*_0_ compared with the other two bands. The lower energy level of the π* band can be explained in terms of the in-plane tensile strain in the films^28^. The π* band (band 3) is formed by antibonding vanadium *d* and oxygen *p* states. In the present films, the in-plane lattice is increased by the lattice matching between film and TiO_2_(001) substrate. Such an in-plane tensile strain increases the V-O bond distance, thus decreases the *p*-*d* overlap. This can decrease the energy level of the π* (band 3) bands relative to those of the *d*_||_ bands. We determined the *d*_||_ bands in the Γ–*X* direction. The next step is to elucidate the FS topology around Γ in the Γ*XZR* plane for the metallic phase of the VO_2_ thin films.

**Figure 5:**
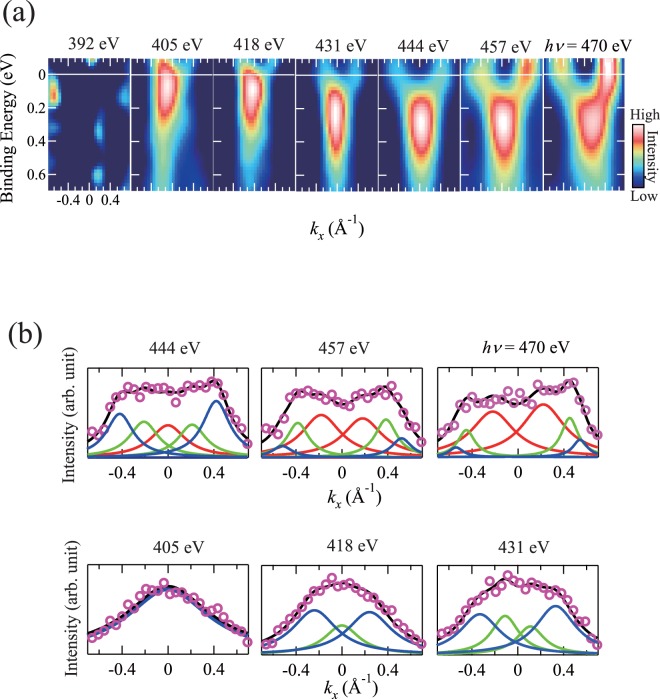
(**a**) Laplacian of the ARPES intensity plot measured at incident photon energy values of 392, 405, 418, 431, 444, 457, and 470 eV. (**b**) MDCs at *E*_B_ = 0 eV in the ARPES intensity maps. The results of the spectral fittings of the MDCs are also shown. In the fitting procedure, a Lorentzian function and constant background are used. Each pair of peaks is constrained to have the peak position symmetrical with respect to *k*_x_ = 0 and an equivalent full width at the half maximum. The open circles are experimental data; black lines are the fitting result; and blue, green, and red lines are the components used for the fittings.

ARPES measurements were performed by decreasing the incident photon energy from 470 to 392 eV in the normal emission mode for the metallic phase of the VO_2_ thin films. In Fig. 5(a), the ARPES intensity plots of the Laplacian are shown to emphasize the peak structures. It was observed that the electron bands shift upward systematically with decreasing photon energy. This indicates the presence of the electron surface in the Γ*XRZ* plane. To determine the *k*_F_s of the electron surface, we performed the spectral fitting of MDCs at *E*_B_ = 0 eV for each spectrum. As shown in Fig. 5(b), the MDCs are reasonably reproduced using the three electron bands observed at Γ (*hν* = 470 eV). The *k*_F_ values are determined from the peak position of each pair of peaks. As shown in the figure, the *k*_F_s move to the center position with decreasing photon energy, and then merge at *hν* = 444 eV for band 3, then at 418 eV for band 2, and finally at 405 eV for band 1. The results indicate the presence of three electron surfaces around Γ in the Γ*XRZ* plane for the metallic phase of the VO_2_ thin films. We also performed the spectral fitting of the second-derivative EDCs at Γ and MDCs for each spectrum as in the case of the spectrum at 470 eV, and the results for the spectra at 457 and 431 eV are depicted in Supplementary Fig. S1.

**Figure 6:**
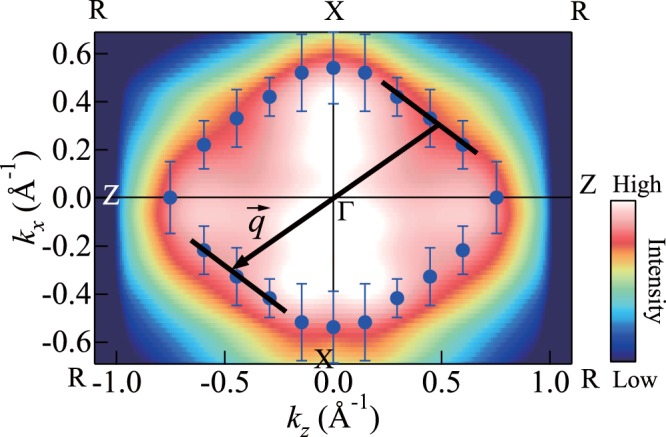
FS mapping in Γ*XRZ* plane for metallic phase of VO_2_ thin films. The *k*_F_s of band 1 (closed blue circles) are superimposed on the figure. The error bars of *k*_F_s are determined from the peak positions where the fitting results do not reproduce the experimental data well (see Supplementary Fig. S2). The band-1 FS topology exhibits flat portions (solid lines). An arrow indicates the nesting vector $\overrightarrow{q}$ = Γ*R*.

Figure 6 shows the FS cross-sections in the Γ*XRZ* plane, which are obtained from the ARPES intensity mapping at *E*_F_ for the metallic phase of the VO_2_ thin films in Fig. 5(a). As shown in the figure, the FS of three electron bands is evident. Among the three surfaces, we focus on the band-1 electron surface to elucidate its topology. By superposing the *k*_F_s of band 1 on Fig. 6, we can observe the FS topology of band 1 clearly. The most noticeable feature of the band-1 FS topology is the presence of flat portions. The flat portions nest into each other when translated by $\overrightarrow{q}$ = Γ*R*. This indicates that the band-1 FS topology possesses the nesting feature corresponding to a nesting vector $\overrightarrow{q}$ = Γ*R*. The magnitude of the nesting vector is |$\overrightarrow{q}$| = |Γ*R*| = 2|$\overrightarrow{k}$_F_| = 1.2 Å^−1^. Our results strongly indicate the formation of the CDW with $\overrightarrow{q}$ = Γ*R* and thus the importance of Peierls transition for the mechanism of the MIT in VO_2_.

**Figure 7:**
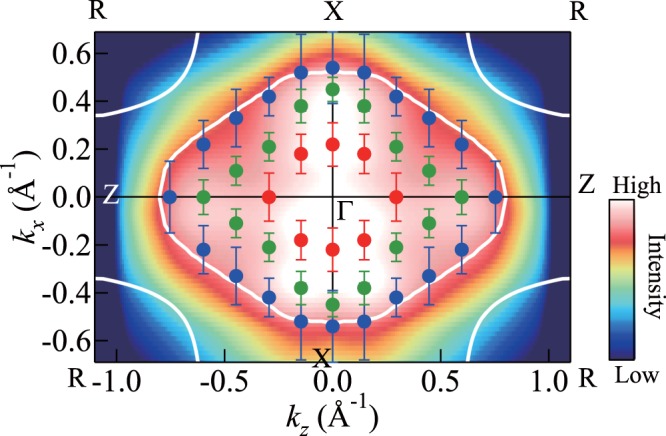
FS mapping in Γ*XRZ* plane for metallic phase of VO_2_ thin films. The *k*_F_s determined from the peak positions in Fig. 5(b) are superimposed on the figure. The closed blue circles correspond to the *k*_F_s of band 1, closed green ones to those of band 2, and closed red ones to those of band 3. The lowest *d*_||_ band FS topology obtained through calculations^17^ is shown for comparison (white lines).

The FS topologies of the three electron bands are shown in Fig. 7, together with the theoretical band-1 FS topology for comparison. The experimental results indicate certain similarities and differences from the calculation. With regard to the similarities, the major contribution to the Fermi surface arises from bands 1 and 2, whereas a small electron pocket around Γ (band 3) is observed in the experiment. Furthermore, the band-1 FS topology in the experiment is largely similar in shape and size to that in the calculation. The difference between the experiment and calculation is with regard to the electron surface around *R*. The electron surface around *R* is not observed in the experiment, whereas it is manifested in the calculation. However, at this stage, we should observe caution while forming conclusions. It is considered likely that the size of the electron surface around *R* becomes small because of the compensation by the small size of the electron surface around Γ. In the present work, we observe the FS within the *k*_z_ range of ± 0.75 Å^−1^ and do not observe the FS in the whole range of *k*_z_ in the Γ*XRZ* plane. In order to form a conclusion, it is necessary to perform detailed ARPES measurements around *R* and determine the overall shape of the FS in the Γ*XRZ* plane.

Finally, we wish to mention that a synchrotron radiation ARPES measurement using epitaxial thin films is a promising method for determining the band structures and FS topology of VO_2_. The experimentally determined band structures and FS topology render it feasible to compare with the theoretical calculations. This will aid the understanding of the origin of the MIT and elucidate an effective model for the MIT in VO_2_. For example, according to the calculation^17^, the similar nested FS is predicted in the direction parallel to the *xz* plane in a short range, resulting in the formation of a cylinder with small height along the *y*-axis. This indicates that the nested FS is limited within a short range along the *y*-axis. It would be worthwhile to examine this prediction by the synchrotron radiation ARPES study using VO_2_ thin films. The results will provide further information for an understanding of the mechanism of the transition in VO_2_. We conjecture that the synchrotron radiation ARPES study using epitaxial thin films will provide crucial information to understand the underlying physics of the MIT in VO_2_.

## Conclusions

We determined the band structures and FS topology of the metallic phase of VO_2_ by performing synchrotron radiation ARPES measurements of VO_2_/TiO_2_(001) epitaxial thin films. The ARPES studies reveal the presence of three electron pockets around Γ in the Γ–*X* direction. Among the three, the two lower electron pockets can be attributed to *d*_||_ bands and the higher pocket to a π* band. It is also revealed that these three bands form three electron surfaces around Γ in the Γ*XRZ* plane. The most crucial observation is that the FS topology of the lowest *d*_||_ band includes the nesting feature with a nesting vector $\overrightarrow{q}$ = Γ*R*. The present FS topology exhibits shape and size similar to that predicted, whereas the predicted electron surface around *R* is not observed. The present results strongly indicate the formation of the CDW with $\overrightarrow{q}$ = Γ*R* and thus the significant contribution of Peierls transition to the MIT in VO_2_.

## Methods

### Sample preparation and characterization

The methods of the film preparation and characterization followed the previous work^21^. VO_2_ thin films were grown on TiO_2_(001) single crystal substrates using a PLD technique with KrF laser (*λ* = 248 nm). V_2_O_3_ was used as a target. During the deposition, the substrate temperature was maintained at 653 K, and oxygen pressure was maintained at 0.9 Pa. The deposition time was 0.5 h with a repetition rate of 1 Hz and 1 h with a repletion rate of 3 Hz. After deposition, the films were cooled down to 300 K at the same oxygen pressure. The laser fluence was 1.5 J/cm^2^. The film thickness was measured to be 11 nm by a profilometer. The prepared films were examined by X-ray diffraction (XRD) measurements using Cu *Kα* radiation (Rigaku RINT-2000/PC). The surface morphology of the films was examined by atomic force microscopy (AFM, SPA400 + SPI3800N, Seiko Instruments). The resistivity measurements were carried out using a standard four-point probe method in a physical properties measurement system (Quantum Design).

### Synchrotron radiation angle-resolved photoemission spectroscopy (ARPES)

Synchrotron radiation ARPES measurements were performed on BL-2C of Photon Factory at KEK using linearly polarized light. In the present measurements, the (001) surface of the VO_2_ thin film was situated normal to the analyzer, and the [100] direction of the rutile-type tetragonal phase was set to be parallel to the polarization direction in order to obtain the ARPES spectra along the *k*_x_ direction. The ARPES spectra were measured at 380 K for the metallic phase and at 250 K for the insulator phase under an ultrahigh vacuum of ~10^−8^ Pa using a Gammadata-Scienta SES2002 spectrometer with an acceptance angle of ± 1°. The FS in the Γ*XRZ* plane was determined using the ARPES measurements along the *k*_x_ direction combined with those along the *k*_z_ direction. The ARPES spectra along the *k*_z_ direction were captured by changing the excitation photon energy *hν* from 392 to 470 eV. The region of these photon energies correspond to the 6^th^ brillouin zone of the rutile-type tetragonal structure in the metallic phase. We provide a short explanation of the normal emission ARPES measurements. This explanation has been described in the previous work^25^. In the ARPES measurement, we view the exciting process in the direct transition picture. In a direct transition, it is assumed that the momentum of an electron excited from one band to another band is unchanged. Thus,

$$\begin{array}{rcl}{E}_{{\rm{f}}} & = & {E}_{{\rm{i}}}+hv,\\ {k}_{{\rm{f}}} & = & {k}_{{\rm{i}}},\end{array}$$

where *E*_f_ and *E*_i_ are the final- and initial-state energies of the electron in the solid, *hν* is the photon energy, and *k*_f_ and *k*_i_ are the final- and initial-state wave vectors, respectively. When we assume a free-electron final state inside the crystal, we obtain

$${E}_{{\rm{f}}}({k}_{{\rm{f}}})={{\hbar }}^{2}{{k}_{{\rm{f}}}}^{2}/2m\,-\,{V}_{0}({V}_{0} > 0),$$

where *m* is the mass of the electron and *V*_0_ is a constant positive inner potential referenced to the vacuum level which defines the zero of the free-electron final-state band. Because the component of *k*_f_ parallel to the surface, *k*_f//_, is conserved to within a surface reciprocal-lattice vector upon exciting the solid, when only electron emitted normal to the surface are detected, we have

$$\begin{array}{lllll}{k}_{{\rm{i}}//} & = & {k}_{{\rm{f}}{\parallel }} & = & 0,\\ {k}_{{\rm{i}}\perp } & = & {k}_{{\rm{f}}\perp } & = & 0.51{(E+{V}_{0})}^{1/2}{\AA }^{-1},\end{array}$$

where *k*_i//_ is the component of *k*_i_ parallel to the surface, free-electron mass is assumed, and *E* and *V*_0_ are expressed in eV. Thus, as the photon energy is varied, we obtain different values of *k*_i⊥_ and can measure the electronic state along a direction in *k* space normal to the surface. When the momentum of the photon is taken into consideration, the above equations for energy dispersion are modified as follows:

$$\begin{array}{rcl}{k}_{{\rm{i}}//} & = & -{k}_{{\rm{p}}//},\\ {k}_{{\rm{i}}\perp } & = & 0.51{(E+{V}_{0})}^{1/2}-{k}_{{\rm{p}}\perp }{\AA }^{-1},\end{array}$$

where *k*_p//_ is the surface parallel momentum component of the photon and *k*_p⊥_ is the surface normal momentum component of the photon. A *V*_0_ of 16 eV was used for the present work^25^. The total energy resolution was approximately 150 meV. The Fermi level (*E*_F_) position was determined by measuring gold spectra. The deviation of the photon energy was estimated from the *E*_F_ position before and after taking the spectra to be within ± 100 meV.

## Electronic supplementary material

Supplementary Information
