# Supplementary material for: Fermi surface topology in a metallic phase of VO2 thin films grown on TiO2(001) substrates
Source: Sci Rep. 2018 Dec 17;8:17906. doi: 10.1038/s41598-018-36281-8 (PMC6297140; doi:10.1038/s41598-018-36281-8)
Supplement: Supplementary file 1 — Supplementary Information [file 41598_2018_36281_MOESM1_ESM.pdf]

Supplementary Information

Fermi surface topology in a metallic phase of VO_2 thin films grown on TiO_2(001) substrates

Yuji Muraoka[1,*], Hiroki Nagao[2], Yuichiro Yao[2], Takanori Wakita[1], Kensei Terashima[1], Takayoshi Yokoya[1], Hiroshi Kumigashira[3,§] & Masaharu Oshima[4]

[1]Research Institute for Interdisciplinary Science, Okayama University, 3-1-1 Tsushima-naka, Tsushima, Kita-ku, Okayama 700-8530, Japan

[2]Graduate School of Natural Science and Technology, Okayama University, 3-1-1 Tsushima-naka, Tsushima, Kita-ku, Okayama 700-8530, Japan

[3]High Energy Accelerator Research Organization (KEK), Photon Factory, 1-1 Oho, Tsukuba, Ibaraki 305-0801, Japan

[4]The Institute for Solid State Physics, The University of Tokyo, 5-1-5 Kashiwanoha, Kashiwa, Chiba 277-8581, Japan

[§]Present address: Institute of Multidisciplinary Research for Advanced Materials, Tohoku University, 2-1-1 Katahira, Aoba-ku, Sendai 980-8577, Japan

[*]Corresponding author: ymuraoka@cc.okayama-u.ac.jp

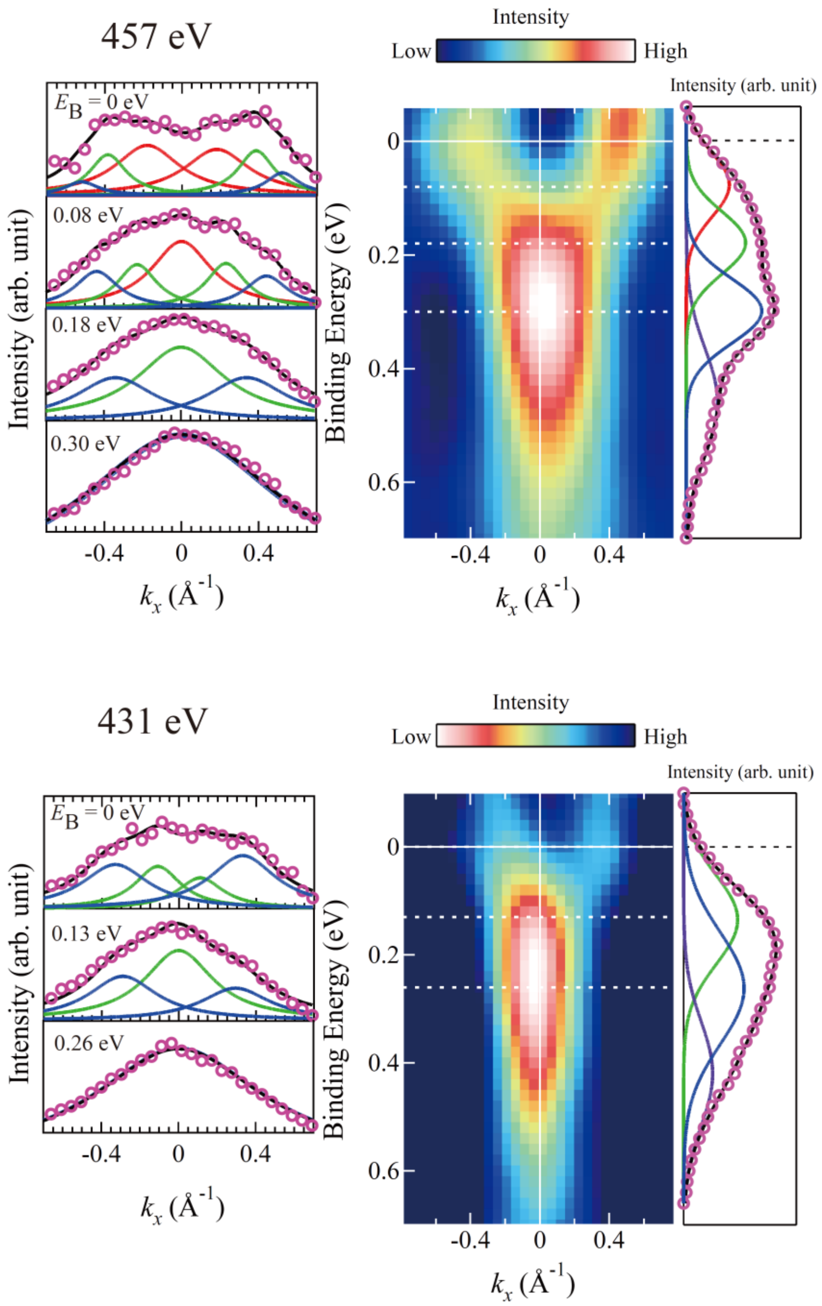

Fig. S1. Results of the spectral fitting at 457 and 431 eV. (Middle) Experimental band structure in the k_x direction of $VO_2/TiO_2(001)$. (Right) Second-derivative ARPES spectra with respect to the EDCs at Γ of $VO_2/TiO_2(001)$. The open circles are experimental data, black line represents the fitting result, and lines colored other than black represent the component used for the fitting. (Left) MDCs at different binding energies of ARPES spectra for $VO_2/TiO_2(001)$. The open circles are experimental data; black lines are the fitting result; and blue, green, and red lines are the components used for the fittings.

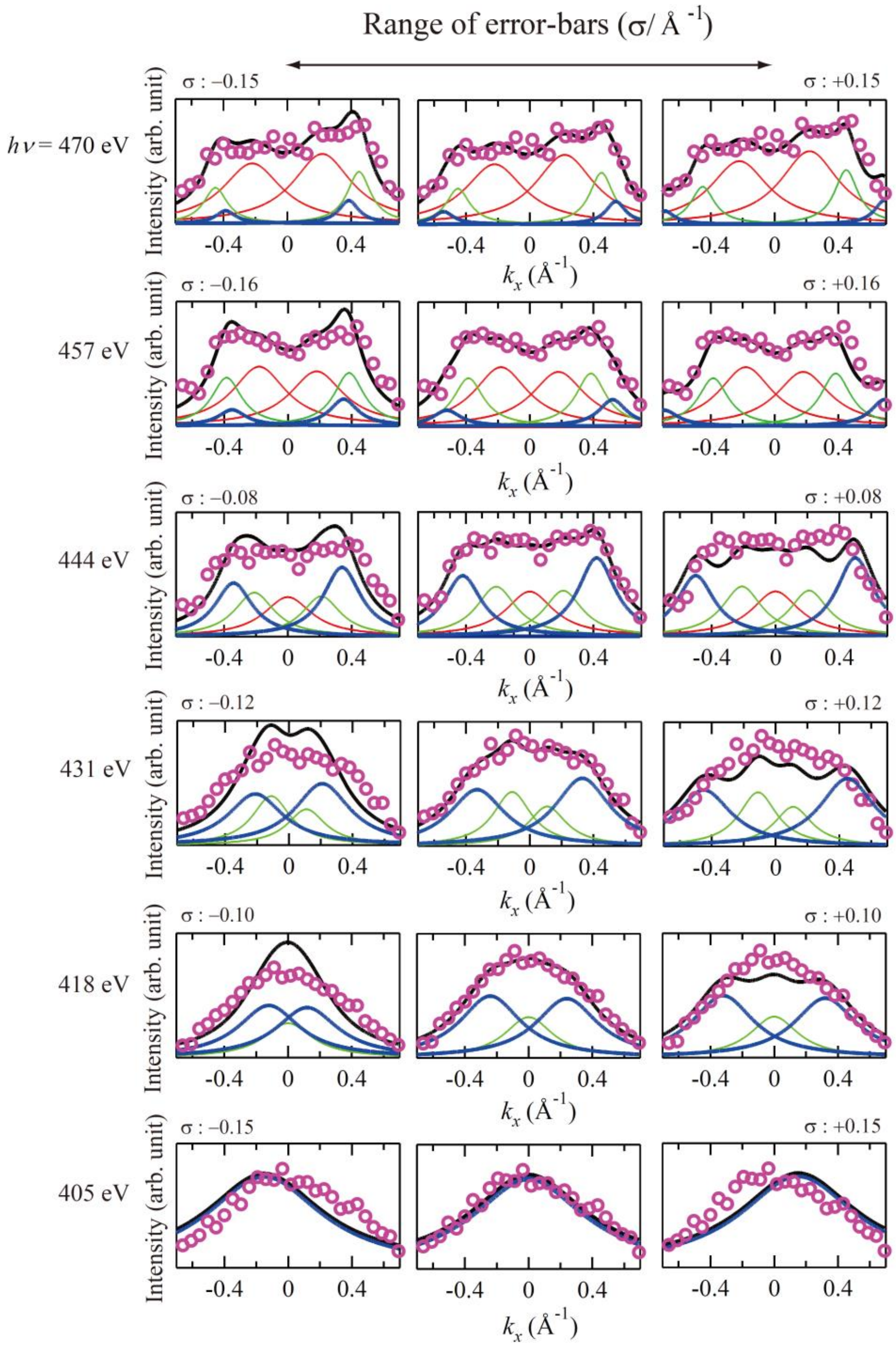

Fig. S2. Results of the spectral fitting of MDCs at E_B = 0 eV for the band 1 at different photon energies. The error bars of k_Fs are determined from the peak positions where the fitting results do not reproduce the experimental data well. In the fitting procedure, the components of bands 2, and 3 are fixed and are not changed for all the photon energies. The open circles are experimental data; black lines are the fitting result; and blue, green, and red lines are the components of band 1, 2, and 3, respectively.
